# An Exploration of Deep-Learning Based Phenotypic Analysis to Detect Spike Regions in Field Conditions for UK Bread Wheat

**DOI:** 10.34133/2019/7368761

**Published:** 2019-07-31

**Authors:** Tahani Alkhudaydi, Daniel Reynolds, Simon Griffiths, Ji Zhou, Beatriz de la Iglesia

**Affiliations:** ^1^University of East Anglia, Norwich Research Park, Norwich NR4 7TJ, UK; ^2^University of Tabuk, Faculty of Computers & IT, Tabuk 71491, Saudi Arabia; ^3^Earlham Institute, Norwich Research Park, Norwich NR4 7UZ, UK; ^4^John Innes Centre, Norwich Research Park, Norwich NR4 7UH, UK; ^5^Plant Phenomics Research Center, China-UK Plant Phenomics Research Centre, Nanjing Agricultural University, Nanjing 210095, China

## Abstract

Wheat is one of the major crops in the world, with a global demand expected to reach 850 million tons by 2050 that is clearly outpacing current supply. The continual pressure to sustain wheat yield due to the world's growing population under fluctuating climate conditions requires breeders to increase yield and yield stability across environments. We are working to integrate deep learning into field-based phenotypic analysis to assist breeders in this endeavour. We have utilised wheat images collected by distributed CropQuant phenotyping workstations deployed for multiyear field experiments of UK bread wheat varieties. Based on these image series, we have developed a deep-learning based analysis pipeline to segment spike regions from complicated backgrounds. As a first step towards robust measurement of key yield traits in the field, we present a promising approach that employ Fully Convolutional Network (FCN) to perform semantic segmentation of images to segment wheat spike regions. We also demonstrate the benefits of transfer learning through the use of parameters obtained from other image datasets. We found that the FCN architecture had achieved a Mean classification Accuracy (MA) >82% on validation data and >76% on test data and Mean Intersection over Union value (MIoU) >73% on validation data and and >64% on test datasets. Through this phenomics research, we trust our attempt is likely to form a sound foundation for extracting key yield-related traits such as spikes per unit area and spikelet number per spike, which can be used to assist yield-focused wheat breeding objectives in near future.

## 1. Background

As one of the world's most important cereal crops, wheat is a staple for human nutrition that provides over 20% of humanities calories and is grown all over the world on more arable land than any other commercial crops [1]. The increase of population, rapid urbanisation in many developing countries, and fluctuating climate conditions indicate that the global wheat production is expected to have a significant increase in the coming decades [2]. According to the Food & Agriculture Organisation of the United Nations, the world's demand for cereals (for food and animal feed) is expected to reach 3 billion tonnes by 2050 [3]. Nevertheless, it is critical that this increase of crop production is achieved in a sustainable and resilient way, for example, through deploying new and useful genetic variation [4]. By combining suitable genes and traits assembled for target environments, we are likely to increase yield and yield stability to address the approaching global food security challenge [5].

One effective way to breed resilient wheat varieties in fluctuating environmental conditions to increase both yield and the sustainability of crop production is to screen lines based on key yield-related traits such as the timing and duration of the reproductive stage (i.e., flowering time), spikes per unit area, and spikelet number per spike. Based on the performance of these traits, breeders can select lines and varieties with better yield potential and environmental adaptation [6–8]. However, our current capability to quantify the above traits in field conditions is still very limited. The trait selection approach still mostly depends on specialists' visual inspections of crops in the field as well as their evaluation of target traits based on their experience and expertise of the crop, which is labour-intensive, relatively subjective, and prone to errors [9, 10]. Hence, how to utilise computing sciences (e.g., crop imaging, computer vision and machine learning) to assist the wheat breeding pipeline has become an emerging challenge that needs to be addressed.

With rapid advances in remote sensing and Internet-of-Things (IoT) technologies in recent years, it is technically feasible to collect huge amounts of image- and sensor-based datasets in the field [11, 12]. Using unmanned aerial vehicles (UAVs) or fixed-wing light aircrafts [13–15], climate sensors [16], ground-based phenotyping vehicles [17, 18], and/or large in-field gantry systems [19, 20], much crop growth and development data can be collected. However, new problems have emerged from big data collection, which include the following: (1) existing remote sensing systems cannot locate the right plant from hundreds of plots, at the right time; (2) it is not possible to capture high-frequency data (e.g., with a resolution of minutes) to represent dynamic phenological traits (e.g., at booting and anther extrusion stages) in the field; (3) how to extract meaningful phenotypic information from large sensor- and image-based data; (4) traditional computer vision (CV) and machine learning (ML) are not suitable for carrying out phenotypic analysis for in-field plant phenotyping datasets, because they contain large variations in quality and content (e.g., high-dimensional multispectral imagery) [21–23]. Hence, many breeders and crop researchers are still relying on the conventional methods of recording, assessing, and selecting lines and traits [24–27].

The emerging artificial intelligence (AI) based robotic technologies [28–30] and distributed real-time crop phenotyping devices [31, 32] have the potential to address the first two challenges as they are capable of acquiring continuous visual representations of crops at key growth stages. Still, the latter two challenges are more analytically oriented and require computational resolutions to segment complicated backgrounds under changeable field lighting conditions [33, 34]. As a result, ML-based phenotypic analysis is becoming more and more popular in recent years. Some representative approaches that use CV and ML for traits extraction in plant research are as follows: PhenoPhyte [35] uses the OpenCV [36] library to segment objects based on colour space and adaptive thresholding, so that leaf phenotypes can be measured; PBQuant [37] employs the Acapella™ library to analyse cellular objects based on intensity distribution and contrast values; MorphoLeaf [38], a plug-in of the Free-D analysis software, performs morphological analysis of plants to study different plant architectures; BIVcolor [39] uses a one-class classification framework to determine grapevine berry size using the MATLAB's Image Processing Toolbox; Phenotiki [40] integrates off-the-shelf hardware components and easy-to-use Matlab-based ML package to segment and measure rosette-shaped plants; Leaf-GP [41] combines open-source Python-based image analysis libraries (e.g., Scikit-Image [42]) and the Scikit-Learn [43] library to measure growth phenotypes of* Arabidopsis* and wheat based on colour, pattern, and morphological features; state-of-the-art deep learning (e.g., Convolutional Neural Network, CNN) has been employed to carry out indoor phenotyping for wheat root and shoot images using edge- and corner-based features [44]; finally, recent advances have been made in the application of deep learning to automate leaf segmentation and related growth analysis [45, 46].

Most of the above solutions rely on relatively high-clarity images, when camera positions are fixed and lighting conditions are stable; however, it is not possible to reproduce imagery with similar quality in complicated field conditions, where yield-related traits were assessed. For this reason, we have explored the idea of isolating regions of interest (ROI, i.e., spike regions) from noisy background so that sound phenotypic analysis could be carried out. Here, we describe the approach of applying a Fully Convolutional Network (FCN) [47] to segment spike regions from wheat growth images based on annotated image data collected by CropQuant (CQ) field phenotyping workstations [32]. The target traits can be seen in Supplementary Figure 1, for which we have utilised the transfer learning approach to load ImageNet [48, 49] parameters to improve the performance of the learning model. In addition, we investigated the effects of two input image sizes when training the FCN, as well as the model's performance at each key growth stage.

To our knowledge, the FCN approach has not been applied to classify spike regions in field conditions. The result of our work is based on three-year wheat image series, which is highly correlated with ground truth data manually labelled. Furthermore, through the evaluation of outputs of each max-pooling layer in the learning architecture, novel vision-based features can be derived to assist crop scientists to visually debug and assess features that are relevant to the trait selection procedure. We believe that the methodology presented in this work could have important impacts on the current ML-based phenotypic analysis attempts for segmenting and measuring wheat spike regions. The phenotypic analysis workflow concluded in our work is likely to form a reliable foundation to enable future automated phenotypic analysis of key yield-related traits such as spike regions, key growth stages (based on the size of detected spike regions), and spikelets per unit area.

## 2. Methods

### 2.1. Wheat Field Experiments

To assess key yield-related traits for UK bread wheat, we have utilised four near isogenic lines (NILs) of bread wheat in field experiments, representing genetic and phenotypic variation with the similar genetic background called “Paragon”, an elite UK spring wheat that is also used in the Biotechnology and Biological Sciences Research Council's (BBSRC) Designing Future Wheat (DFW) Programme. The four NILs include Paragon wildtype (WT),* Ppd* (photoperiod insensitive), and* Rht* genes (semidwarf) genotypes cloned at John Innes Centre (JIC) [50, 51], which were monitored by distributed CQ workstations in real field environments and measured manually during the key growth stages in wheat growing seasons from 2015 to 2017.

### 2.2. Image Acquisition

The Red-Green-Blue (RGB) image series used in this study were collected from 1.5-metre-wide (5-metre-long) wheat plots during a three-year field experiment. To generate continuous vision representation of key growth stages of the crop in the field, four CQ workstations were dedicated to conduct high-frequency (one image per hour) and high-resolution (2592x1944 pixels) imaging in order to acquire target yield-related traits expression. Between May and July in three growing seasons (i.e., covering booting, GS41–GS49, to grain filling stages, GS71–GS77), over 60 GB image datasets have been generated by CQ devices. For each growing season, 30 representative images were selected for the deep-learning based phenotypic analysis.

**Figure 1:**
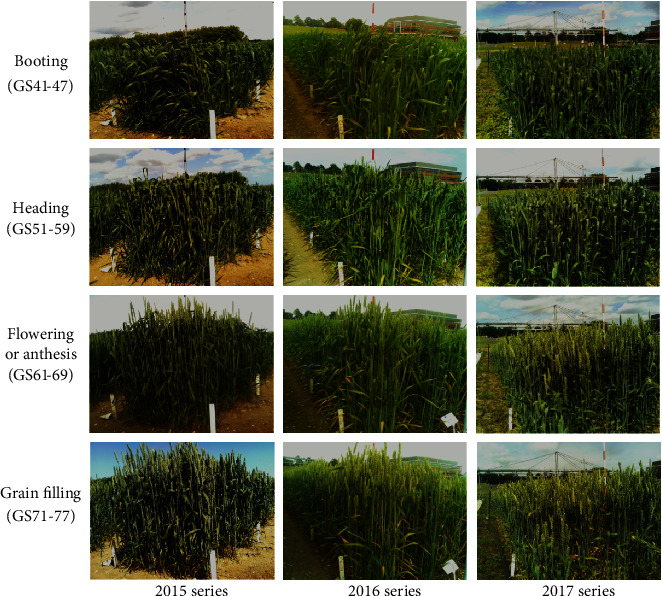
Wheat growth image series collected by CropQuant workstations, from 2015 growing season to 2017 growing season, ranging from booting to grain filling stages.

In order to maintain similar contrast and clarity of wheat images in varied lighting conditions in the field, the latest versions of open-source* picamera* imaging library [52] and Scikit-image [42] were employed to automate the adjustment of white balance, exposure mode, shutter speed, and calibration during the image acquisition. In-field image datasets were synchronised with centralised storage at Norwich Research Park (NRP) using the Internet-of-Things based CropSight system [53].  Figure 1 shows the wheat plot images acquired by CQ workstations from 2015 to 2017 (in columns), indicating that images selected for the yield-related traits analyses were under varying in-field illumination conditions and weather conditions, containing a range of background objects during the experiments.

### 2.3. Wheat Growth Datasets for Training, Validation, and Testing

**Figure 2:**
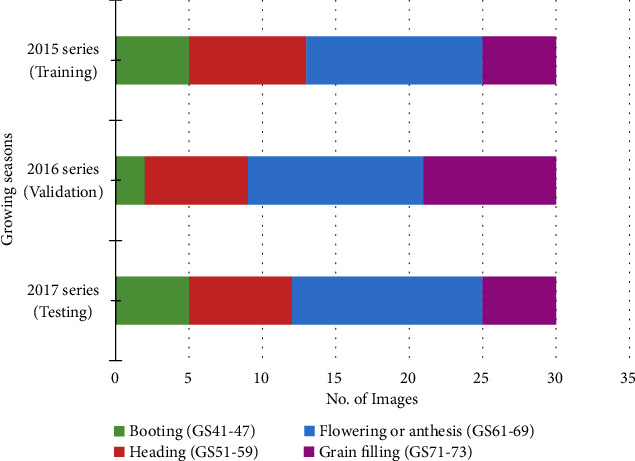
The distribution of selected images in each growth stage collected in three-year field experiments, which are used for training, validation, and testing when establishing the deep-learning architecture.

Because images were collected from three consecutive years that cover four key growth stages (Figure 1), we decided to use the 2015 dataset to train the models, because of the constant clarity and contrast of the image series. Then, we use the 2016 dataset to validate our learning model and the final year, i.e., the 2017 dataset, to test the model. This training strategy gives us a reasonably robust validation of the performance of our model as the unseen dataset in 2017 can be utilised to test the generalisation of the model.  Figure 2 illustrates the distribution of selected images in each growth stage in each growing season (30 images per year, 90 in total). Amongst these datasets, the flowering stage has the highest number (37 out of 90), followed by ear emergence (22 images), grain filling stages (19 images), and booting (12 images). The reason for this arrangement is that the flowering stage represents the phase when spikes are fully emerging, whereas wheat spikes are normally partially hidden at booting and heading stages (i.e., GS41-59 [8]). It is worth noting that the 2015 dataset does not contain many booting images due to the short-term nature of wheat booting, which normally finishes within 1-2 days. Hence, it is an interesting test case for us to train a deep-learning model that can segment spike regions collected in multiple years during the process of ear emergence (e.g., spikes have partially emerged) under challenging in-field lighting conditions.

### 2.4. The Workflow for Training and Testing

**Figure 3:**
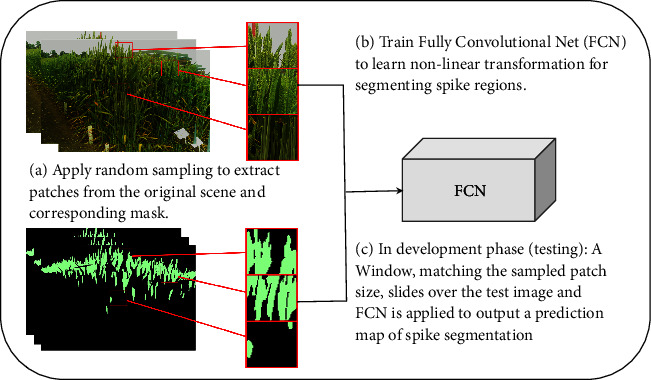
The training, validation, and testing strategy for developing Fully Convolutional Networks (FCN). (a) The selection of subimages for manually labelling spike regions, (b) training an FCN to segment spike regions with the manual labelled data, and (c) performing model testing at the image level to predict spikes.

We randomly sampled subimages from the original images for training and testing.  Figure 3 explains a high-level workflow that we followed, including the selection of subimages for wheat growth image series, manually labelling spike regions at the image level (Figure 3(a)), training a FCN with manual labelled data (Figure 3(b)), and performing model testing at the image level for predicting spike regions (Figure 3(c)). Similar to standard convolutional neural network approaches, a sliding window is used to validate performance on the 2016 and then test on the 2017 dataset. We experimented with two sliding windows (512×512 and 128×128 pixels) together with a fixed stride of* s* to create predictions of wheat spike regions in each window. The window size corresponds to the subimage size that is chosen by experimental setting. The result of the workflow is a prediction map with size w × h × cl, where w and h correspond to the original image's width and height and cl is the number of classes, two in our case. Results from experimentation on different sizes of the sliding window are discussed in Result section.

### 2.5. Fully Convolutional Network

We applied the FCN approach for our semantic segmentation problem, in particular FCN-8 due to its enhanced results for similar problems. FCN associates each pixel with a specific class label. The novelty and advantage of applying FCN in this study is that it transforms the nonspatial output produced by the deep classifier to a spatial one that is required during the semantic segmentation task. This is accomplished through transforming the fully connected layers attached at the end of the deep classifier, so that image level prediction can be produced. Fully convolutional layers that replace fully connected layers can preserve the spatial information of target objects and hence enable the pixel level prediction [47]. This approach provides a solution to localise and detect targeted objects based on manually labelled training datasets constructed in previous steps. However, the output of the FCN at this stage has a lower resolution than the original input image and yields a coarse output. To tackle this down sampling problem, FCNs were proposed to reverse the effect of repetitive subsampling through upsampling [54]. The upsampling method is based on backward convolution (also called deconvolution). Furthermore, FCN provides another enhancement by applying a concept called skip connection (see detailed explanation below). This takes advantage of the hierarchy resulting from any convolutional neural network that starts with local feature maps describing the finest information (i.e., edges, contrast, etc.) and ends with the coarsest information that describe the semantics of the target objects (i.e., the more generic features of the region). The FCN combines those levels to produce a more detailed output map.

### 2.6. Learning Architecture

**Figure 4:**
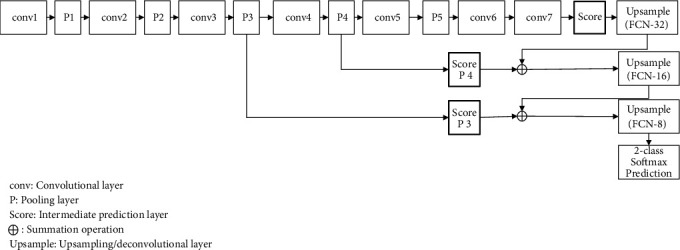
The FCN-8 learning architecture used for segmenting wheat spike regions.

The learning architecture of the FCN model established for segmenting spike regions is presented in Figure 4, which consists of four components:

1. Very deep convolutional network: the first component of FCN is the so-called very deep convolutional network (VGG 16-layer net, VGG16 [54]). The segmentation-equipped VGG net (FCN-VGG16 or VGG16) has outperformed other classifiers such as AlexNet [49] and GoogLeNet [55] when it was selected as the base for FCN. It is a CNN classifier that achieved the first and second places in the ImageNet localisation and classification competition. Therefore, we have selected VGG16 as the base classifier for the task of spike segmentation. It has 12 convolutional layers arranged in five increasing convolutional depth blocks (Figure 4): (1) the first block, conv1, consists of two convolutional layers with a depth (number of filters) of 64; (2) the second block, conv2, consists of two convolutional layers with a depth of 128; (3) the third block, conv3, consists of three convolutional layers with a depth of 256; and (4) the fourth and fifth blocks, conv4 and conv5, respectively, consist of three convolutional layers with a depth of 512. After each convolutional layer, there is a rectification nonlinearity layer (ReLU) [56]. The filter size selected for all convolutional layers is 3 × 3 with a stride of 1. The reason for choosing such a small receptive field is that a nonlinearity layer can be followed directly to make the model more discriminative [54]. After each block, a max-pool layer is added with a pooling size of 2 × 2 with a stride of 2. There are three fully connected layers at the end of the classifier. The first two fully connected layers, FC6 and FC7, have a depth (units) of 4,096, which are replaced by convolutional layers (conv6 and conv7). The depth of the last connected layer is 1000, which corresponds to the number of classes in the ImageNet competition. The sixteenth (last) layer is the softmax prediction layer, which comes after the last connected layer. It is worth noting that the last connected layer is removed in our architecture as our task requires prediction for two and not 1000 classes.
2. Fully convolutional layers: the second component of FCN is replacing the first two fully connected layers FC6 and FC7 in VGG16 with two convolutional ones (conv6 and conv7). This setting is designed to restore the spatial information of spike regions on a given image.
3. Deconvolutional layers and feature fusion: even though restoring the spikes' spatial details can help with the segmentation task that involves predicting dense output, the output from the fully convolutional layers is still coarse due to the repeat application of convolutions and subsampling (max-pool), which reduces the output size. In order to refine the coarse output and retain the original resolution of the input, the model fuses the learned features from three positions in VGG16 with the upsampling layers. Upsampling or deconvolutional layers reverse the effect of the repetitive application of subsampling and convolving by learning backward convolution. In order to apply the fusion operation, three prediction layers were added: (1) after the last fully convolution layer FC7, (2) after the fourth max-pool P4, and (3) after the third max-pool P3. The reason for predicting at different positions is to fuse lower level information obtained from the lower layers together with higher-level information obtained from the higher layers, which can further refine the output. Next, the output of the first prediction layer is upsampled by applying the first deconvolutional layer. Then, the first upsampled output (FCN-32) is fused with the second prediction layer (Score P4) by applying element-wise summation, where the first skip connection occurs. It is worth noting that a cropping operation is applied to the upsampled output, so that it matches the size of the second prediction output. Then, the output would be upsampled using the second deconvolutional layer (FCN-16) to be fused with the output of the last prediction layer (Score P3), where the second skip connection occurs. Lastly, a final deconvolutional operation is applied to the output to be upsampled to the input size of the original image (FCN-8), as FCN-8 can obtain better results than FCN-16 and FCN-32 due to its recovery of more boundary details through fusing features during skip connections.
4. Softmax layer: the last layer of FCN is a 2-class softmax [57] calculating the probability of each pixel for each class. In our case, two classes (i.e., spike region and background) have been computed.

### 2.7. Cost Function

According to any common semantic segmentation task [47], for each pixel x_ij_ in an image I with a size of* h × w × d*, a corresponding pixel label class* t*_*j*_ from a probability distribution {0,1} is assigned. The predicted class of a certain pixel* y*_*ij*_ is the outcome of the last softmax layer, which generates a probability distribution such that* 0 ≤ y*_*ij*_* ≤ 1*. The learning task is to find a set of parameters (i.e., weights) *θ* that, for a particular loss function* l(y*_*ij*_*(x*_*ij*_*, θ))*, will achieve the minimum distance of the probability distribution between the target class t_j_ and the predicted class* y*_*i*_. The cost function used here is cross entropy, L, which calculates the negative log likelihood of the predicted class y_j_:

(1) $$\begin{array}{c} L=-\overset{m}{\underset{j=1}{\sum }}t_{j}\log y_{j} \end{array}$$

where m is the number of classes and in our case is 2, corresponding to spikelet area versus background.

### 2.8. Training Hyperparameters

Hyperparameters need to be initialised before the training process starts. Then, the training algorithm learns new parameters as part of the learning process [57]. Summary the FCN training hyperparameters values used in our study are listed in Table 1, including the following:

(1) Weight *θ* (parameters)/Bias initialisation: it is good practice when training any deep-learning model from scratch to initialise the weights with random values and the bias with 0. We have chosen an initialisation technique [55] that achieves the optimal results when training from scratch. Their technique generates a mean centred normal distribution with standard deviation *σ* equal to $\sqrt{2/n_{l}}$ where n_l_ is the number of inputs in a certain layer *l*.
(2) Dropout rate probability: this parameter serves as a regulariser to reduce the model overfitting [58]. It determines how many units can be deactivated randomly for every training iteration in a certain layer. In our model, two dropout layers, with a value of 0.5 for *p*, are added after every fully convolutional layer FC6 and FC7.
(3) Intermediate nonlinearity unit: this is an essential component in any CNN that focuses on highlighting and emphasising the relevant features of the data and the task. As a default, we have selected Rectified Linear Unit (ReLu) for this parameter which is an element-wise thresholding operation that is applied on the output of the convolutional layer (resulting feature map) to suppress negative values: *F*(*x*) = max(0, x)where *x* is an element in the feature map.
(4) Epochs: this refers to the number of training iteration, which was set to 125-150.
(5) Optimisation algorithm: the weights are updated for every learning iteration using minibatch stochastic gradient descent (SGD) with momentum:
  (2) $$\begin{array}{c} v_{t}=\gamma v_{t-1}+\eta \nabla_{\theta }J\left(\theta \right),\theta =\theta -v_{t} \end{array}$$
see [59].
The initial learning rate was chosen as 0.001 with a decay of 0.0016 for every epoch. The momentum *γ* is the default 0.9 and the selected minibatch is 20.
(6) To investigate the effect of transfer learning, we kept the number of filters and layers while establishing the CNN architecture, because we want to keep all factors (e.g., filters and layers) stable in order to investigate the effect of these factors.

### 2.9. Training and Validation of the Architecture

We have selected the 2015 dataset for training FCN and the 2016 dataset as the validation set to observe if there is overfitting of the model. However, these images have high resolution (2592×1944). It is not computationally viable to train the model directly using these images, even via a powerful GPU cluster (64GB). Furthermore, we expect that less computing power will be available when deploying models. Therefore, we needed to seek a viable approach to balance the computational complexity and learning outcomes. As a result, we randomly sampled subimages and experiment with two different subsizes, 450 images (512×512 pixels) and 8999 subimages (128×128 pixels), with corresponding manual labels. These were used to investigate whether a larger size subimage could result in better detection outcomes.

We have utilised an early stopping technique when training the model. Early stopping allows us to keep a record of the validation learning (e.g., cost and accuracy) for each learning epoch. It is a simple and inexpensive way to regularise the model and prevent overfitting as early as possible [57, 60]. We have selected the validation cost as the metric to observe for early stopping. The maximum epochs for observing the change in validation cost are 20 epochs. In other words, if the validation cost has not been decreased for 20 epochs, the model training will be stopped and the model weights resulting from the lowest validation cost are saved. We have found that the model for all our experimental trials converges after training for 125 to 150 epochs.

In addition to training the FCN from scratch, we wanted to investigate whether the transfer learning approach [61] can produce improvements in the validation accuracy. One of the advantages of using deep segmentation architectures that are built on top of state-of-the-art classifiers is that we can apply transfer learning. Transfer learning can be described as using “off-the-shelf” pretrained parameters obtained from millions of examples in thousands of object categories such as the ImageNet database [48]. These parameters represent a general library of features that can be used for the first layers of any CNN model since the first layers are only capturing the low-level features of objects (corners, borders, etc.). It is then possible to only fit the higher-level layers of the CNN that are more task and data oriented. Therefore, we can initialise the CNN model with the pretrained parameters and proceed with training the higher layers instead of initialising with random values and training from scratch. The application of transfer learning is extremely beneficial when there are limitations in the sample size and/or variation of example datasets as those are essential to train any sound deep architecture. Therefore, for our work, we have loaded the pretrained weights from the ImageNet challenge to the VGG16 and then trained the model with the same hyperparameter settings described previously.

### 2.10. Experimental Evaluation of the Segmentation

We evaluate the performance of FCN on both 2016 and 2017 datasets. The evaluation is conducted to test the segmentation performance of FCN considering multiple experimental setups. For example, the use of pretrained parameters when training the model (transfer learning) is compared with training from scratch and the use of different subimage sizes is also compared. Furthermore, we compared the performance on each growth stage separately as this might discover interesting interconnections between the monitored growth stages that have strong correlation to the grain production. To verify the result of the segmentation, we report the following metrics that are commonly used in semantic segmentation work [47, 49, 62]:

(1) Global Accuracy (GA) measures the total number of pixels that were predicted correctly over all classes divided by the total number of pixels in the image. The GA can be calculated using
  (3) $$\begin{array}{c} GA=\frac{\sum_{i}t_{p\_i}}{n_{p}} \end{array}$$
where ∑_*i*_*t*_*p*_*i*_ is the number of pixels that are predicted correctly for each class *i* and n_p_ is the total number of pixels in a given image.
(2) Mean class Accuracy (MA) is the mean of spike and nonspike region accuracy. The accuracy for each class can be calculated using
  (4) $$\begin{array}{c} ClassAccuracy=\frac{\sum_{i}n_{ii}}{t_{i}} \end{array}$$
where ∑_*i*_*n*_*ii*_ is the number of pixels that are predicted correctly to be of class *i* and *t*_*i*_ is the number of pixels of a certain class *i*.
(3) Mean Intersection over Union (MIoU) is the mean of IoU of each class. MIoU is considered the harshest metric amongst all because of its sensitivity towards methods with a high false positive *f*_*p*_ rate or false negative *f*_*n*_ rate or both:
  (5) $$\begin{array}{c} IoU=\frac{t_{p}}{t_{p}+f_{p}+f_{n}} \end{array}$$
where, *f*_*p*_,  *t*_*p*_, and *f*_*n*_ denote, respectively, false positive, true positive, and false negative predictions. This metric was also used in the VOC PASCAL challenge [63]. In our case, it penalises methods that are more inclined towards predicting a spike region pixel as background or vice versa.

We reported the spike region and background measures separately for two reasons: (1) it is important to observe the model performance to recognise the spike region not the background; (2) it is clear that the ratio of background pixels to the spike region pixels is high, especially in early growth stages (i.e., booting and heading) where fewer or no spikelets can be found at the image level, indicating that some images can exhibit imbalanced class distribution. Consequently, it is important to observe evaluation measurements for both classes in the context of such class imbalances.

## 3. Results

### 3.1. Transfer Learning

The results in Tables 2 and 3 report on the experiments comparing the FCN model trained from scratch with parameters learned from the reported ImageNet classification [49] task on the segmentation of the 2016 and 2017 datasets, respectively.  Table 2 shows that MA and MIoU have been improved by 1.99 % and 3%, respectively, when using the pretrained parameters in the 2016 set. Particularly, the results of spike regions show an increase in both Spike Accuracy and Spike IoU by 3.25 % and 4.98 %.

The results in Table 3 illustrate that MA and MIoU have improved by 5.7 % and 4.9 % in the 2017 set when using the pretrained parameters. Notably, the results of the spike region show an increase in both Spike Accuracy and Spike IoU of 10.39 % and 8.24 %, respectively.

**Figure 5:**
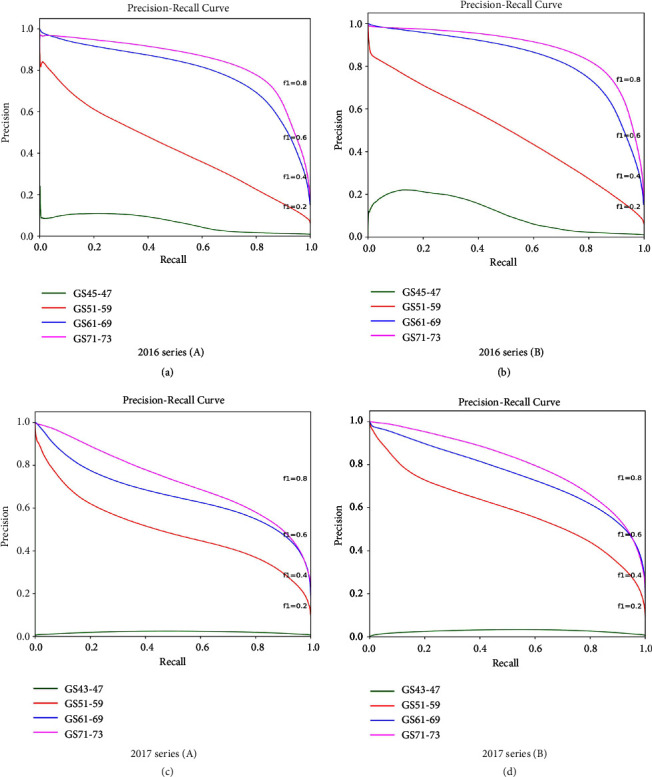
Precision-Recall curves showing the segmentation performance and growth stage curves. (a, b) Training from scratch 2016 series (A) and loading pretrained ImageNet parameters series 2016 series (B) to report the segmentation performance at different monitored growth stages. (c, d) Training from scratch series (A) and loading pretrained ImageNet parameters using series (B) in 2017.

From the results presented in Tables 2 and 3, it is clear that transfer learning has a positive effect on improving performance for both validation and testing datasets. To further verify this finding, we present Precision-Recall curves in Figure 5 for each growth stage for the testing and validation datasets. The left-most subfigures show two graphs that represent the Precision-Recall curves of the models trained from scratch, whereas the right-most graphs represent the curves after loading ImageNet parameters. The top two graphs refer to the 2016 validation dataset, whereas the bottom graphs present results for the 2017 dataset. Although relatively subtle due to the limited sample size, it is noticeable that the transfer learning produces a “lift” effect on the Precision-Recall curves in both years. It is also evident that performance is particularly improved for later growth stages (from flowering and anthesis onwards, when spikes were fully emerged). Given the positive effect of transfer learning, we used this approach in more detailed analyses on different subimage sizes and growth stages.

**Figure 6:**
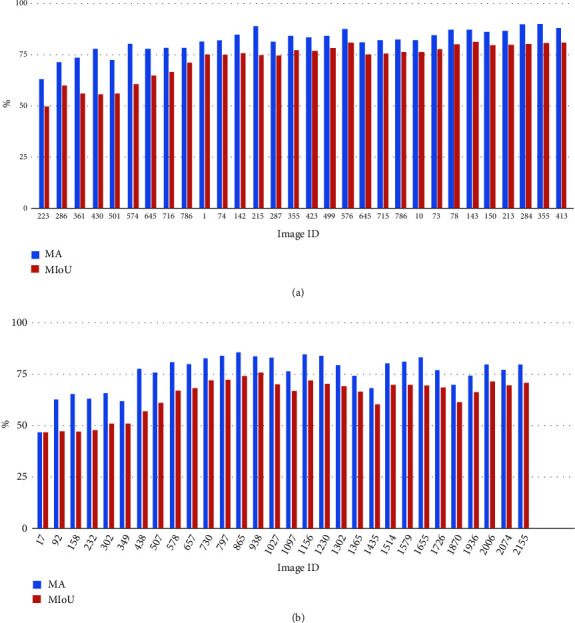
Quantitative results (MA and MIoU) to assess segmentation performance. (a) The 2016 images trained by FCN_8 using pretrained ImageNet parameters. (b) The 2017 image dataset trained by FCN_8 through loading pretrained ImageNet parameters.

Figure 6 shows the segmentation performance using MA and MIoU for the 2016 and 2017 image series when training FCN by loading pretrained ImageNet parameters. The Y-axis represents the values of MA/MIoU (in percentage) and X-axis represents the image ID arranged by its associated growth stage from 2016 to 2017, the smaller ID the earlier growth stage in the growing season (i.e., booting or heading). Figure 6(a) indicates that MA and MIoU are relatively similar in all images, but there is a trend in growth stage as the earlier growth stages achieve lower evaluation metrics scores and the later growth stages achieve higher metrics scores. However, Figure 6(b) does not show a similar trend in 2017; instead, both metrics scores are fluctuating in values across the monitored growth stages. This may indicate that the images in the 2017 series are more challenging, for example, more unexpected objects in the field, less image clarity, and changeable lighting conditions.

### 3.2. Different Subimage Sizes

Tables 4 and 5 illustrate a comparison of two different sets of subimages, 128×128 and 512×512, for spike segmentation on the 2016 and 2017 datasets. In both cases, for almost all measures, the larger subimage sizes produce better performance. For the 2016 set, the MIoU and Spike IoU have increased by 2.68% and 6.9% respectively using the 512x512 subimage size, whereas the MA and Spike Accuracy have improved by 6.03% and 13.55%. For the 2017 set, the MIoU and Spike IoU have increased by 4.3% and 9.9% using the 512x512 subimage size, and the MA and Spike Accuracy have improved by 8.98% and 20%. As a result, we can see that selecting a larger subimage size is likely to lead to better results based on the selected segmentation metrics.

### 3.3. Phenotypic Analysis of Yield and Growth Traits

In Table 6, we report the spike segmentation result according to the growth stages to further investigate FCN's performance for each growth stage in 2016. Note that the 2016 dataset does not contain early or middle booting and hence we could only test late booting. Notably, the model performed very well in both flowering and grain filling stages. For example, in the grain filling stage, the MA and MIoU are 87.12 % and 80.14%, respectively, whereas in the flowering stage, the MA is 84.0% and the MIoU is 77.0 %. In the heading stage, the model has also achieved good results with the MA and MIoU equal to 77.01% and 62.0%. However, FCN has not led to good results in booting, where the MA is 67.6% and IoU is 55.0%. This is not surprising as not enough representative images for this stage were available in the training data.

In Table 7, we report the spike segmentation results based on the wheat growth stages in 2017. The table shows that the model performed well in the flowering stage with the MA equal to 80% and MIoU equal to 69.4%, which is likely achieved due to more imagery data presented in this stage in 2017. The heading stage results and the grain filling stage are similar to the flowering stage. However, the model performed worse on the booting stage, corresponding to the lack of data for this stage in the training set. The results show that FCN performance increases with the development of spikes and it performs better if more representative training data can be included when developing the learning model.

It is worth noting that, for both the 2016 and 2017 results, the GA values for the booting stage are higher compared to the other stages, which is not the case for any other evaluation metrics. This may be caused by the majority of the pixels being background in early growth stages, as those are predicted correctly by the GA metric, which focuses on predicting the sum of pixels regardless of the class. It does, however, reinforce the need for more than one single evaluation metric to assess the fitness of learning models as the GA value may not truly reflect the ability of the model during the segmentation.

### 3.4. Visualisation of FCN Intermediate Activation

**Figure 7:**
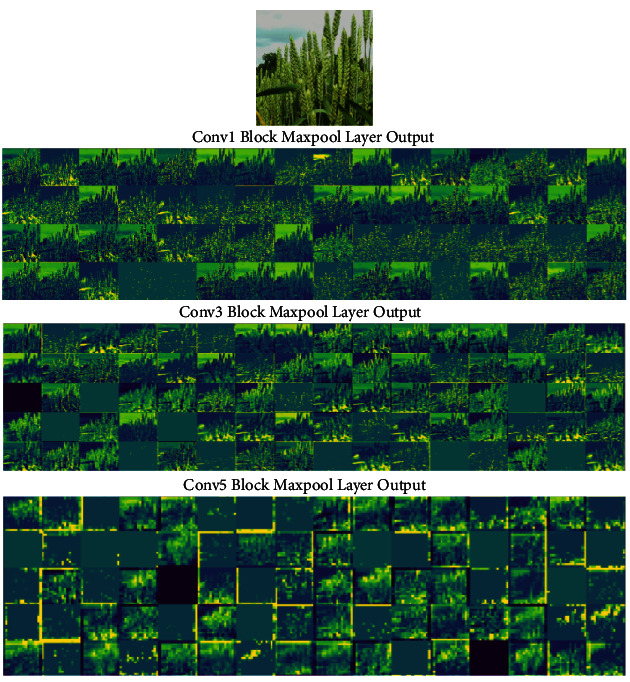
The selection of filters of three intermediate layers (Conv1, Conv3, and Conv5 Block Maxpool outputs) showing activated features that could be used for visually assessing FCN on wheat subimages.

In order to understand and interpret more about the features that FCN is utilising when testing wheat subimages, we have visualised feature maps that are output by each layer in the FCN in the first five blocks (conv1-conv5) [64]. As illustrated in Figure 7, the subimage chosen is from image ID 215 (see supplementary data), which scored the highest spike accuracy amongst all images. To simplify the presentation, we only show a number of feature maps that are output by three layers (i.e., Conv1 Block Maxpool, Conv3 Block Maxpool, and Conv5 Block Maxpool), where regions that are coloured from bright yellow to green indicate where FCN is activated, whereas the darker colour shows regions that are being ignored by the FCN. For example, we can observe that early layers of FCN (Conv1, max-pooling output) are activated by the spikelet-like objects. However, they show very low-level detail information, correlating with the fact that early layers in CNNs capture the lower level of features such as edge and corner-featured objects.

**Figure 8:**
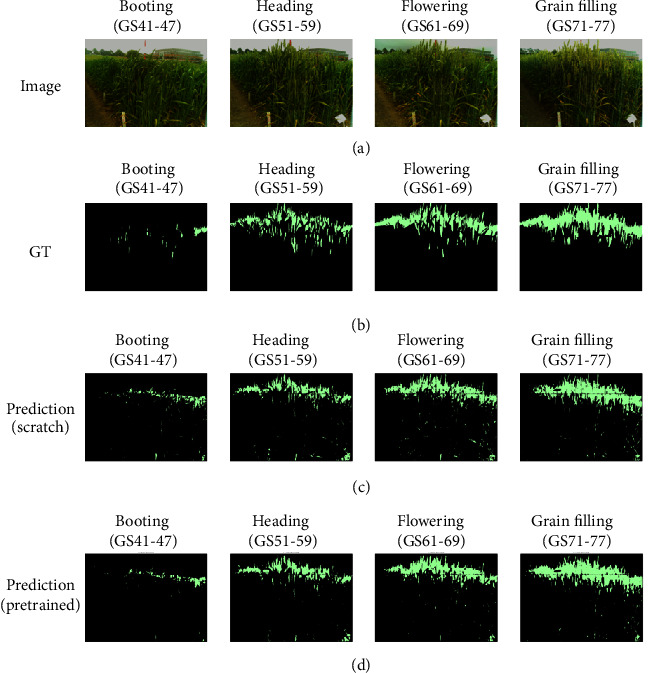
Visualisation of the segmentation result for the 2016 image series. (a) Original image. (b) Ground truth (GT). (c) The result of trained FCN-8 from scratch. (d) The result of trained FCN-8 by loading pretrained ImageNet parameters (from left to right, images were selected to represent different key growth stages).

**Figure 9:**
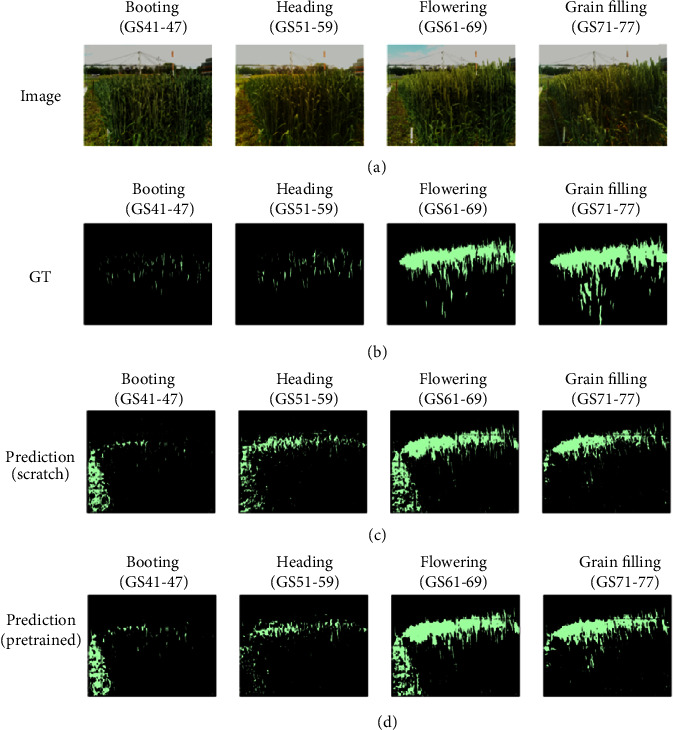
Visualisation of the segmentation result for the 2017 image series. (a) Original image. (b) Ground truth (GT). (c) The result of trained FCN-8 from scratch. (d) The result of trained FCN-8 by loading ImageNet parameters. Images were selected to represent different key growth stages.

The next feature maps (Conv3, max-pooling output) show that the FCN is more focused on the shape and texture-based features, which are considered higher-level abstract features. The last feature maps (Conv5, max-pooling output) shows that the FCN is only preserving the general size- and texture-based features of spike regions as the low-level information has been lost due to repetitive application of pooling operations. In addition, image comparison with original images suggests that the FCN not only recognises spike regions, but also captures other background objects such as sky, soil, and leaves throughout these layers, which leads to segmentation results in Figures 8 and 9.

## 4. Discussion

We have presented a fully convolutional model to perform a complex segmentation task to analyse key yield-related phenotypes for wheat crop based on three-year growth image series. In comparison with many machine learning based indoor phenotypic analysis with ideal lighting and image conditions [65], our work is based on crop growth image series collected in real-world agricultural and breeding situations, where strong wind, heavy rainfall, irrigation and spraying activities can lead to unexpected quality issues. Still, through our experiments, we have proved that the deep-learning approach can lead to promising segmentation performance and the application of transfer learning could result in better spike region segmentation across the monitored key growth stages.

Our work shows that the selection of a larger subimage size (512x512) for the sliding window results in best segmentation performance. This approach translates to higher classification performance (see Tables 2 and 3). In the original FCN research, the algorithm was compared when running on original images and on smaller randomly sampled patches. The conclusion was that the algorithm trained on original images converged faster than on randomly subsampled patches, indicating the bigger images led to better performance. In our case, the subsampled images are comparable in size to the testing images in the original FCN experimentation. When we compare the two subsampling sizes (128x128 and 512x512), smaller subimages results do not contain relevant spike information, which could be the reason why subsampling larger images has led to better results in our work. In addition, it is noticeable that enlarging the perception of the model (i.e., selecting larger input size) was beneficial when learning surrounding objects as it can introduce variation in spike regions such as objects that may appear in subimages during training. This approach has translated to better segmentation performance for our work.

The unique shape of spikes may require more attention around the boundary (see Figures 8 and 9). In many cases, the FCN was successful to some extent in recovering the spike boundary details, which may be due to fusing the features from three locations in the model (conv3-maxpool, conv4-maxpool, and first upsampling layer). The 2015 training dataset was balanced in terms of different weather conditions, from sunny scenes (high exposure of illumination) to rainy and cloudy scenes. The segmentation of spike regions with high and normal lighting conditions was reasonable. However, the model has captured some background objects that were not present in the training dataset such as grass. For example, Figure 9 shows grass regions (to the bottom left of the images) have been wrongly recognised as spike areas. Based on our vision assessment using the method discussed in Figure 7, this error might be caused by severe light exposure, similar colour- and pattern-based features. Again, we believe that more training data could improve the models to avoid such artefacts.

In general, loading pretrained ImageNet parameters (i.e., transfer learning) was beneficial. It has improved the results in 2016 and 2017 sets and also improved the FCN performance for each growth stage (see Precision-Recall curves in Figure 5). Using transfer learning has reduced the false positive rate during the detection of spike regions. This may be because the additional images from ImageNet have enhanced the FCN performance as more examples of different object boundaries and their features are available to the learning algorithm.

As verified in the results section, the FCN has achieved higher accuracy and IoU scores in the later growth stages such as flowering and grain filling. The performance of the FCN was poor in both booting and heading stages and also for spikes partially covered by leaves (Figure 9). The main reason behind this, we believe, is that the distribution of images for different growth stages is unbalanced, with limited booting images represented in the training data. To improve the results of this exploration, more images during booting and heading, when wheat spikes are emerging, will improve the performance of CNN-based models. More importantly, images should be as representative as possible, e.g., including different lighting conditions, variety of background objects, and with different image quality. Furthermore, to address in-field phenotypic analysis challenges caused by image quality (a common problem in real-world field experiments), we suggest that the manually labelled datasets should contain sufficient noise information (e.g., grass and unexpected objects) and regions of interest under varied lighting conditions. When possible, comparisons should be performed within similar crop growth stages as those may be more realistic. Another potential solution is to introduce artificially created images to mimic noise and unexpected objects and add them to the training datasets.

## 5. Conclusions

In this work, we have explored a method that combines deep learning and computer vision to discriminate wheat spike regions on wheat growth images through a pixel-based segmentation. This method was implemented using Python with a TensorFlow backend, which provides the framework for us to establish the FCN architecture. We can then move from the training phase to the final 2-class prediction at the image level. Our goal was to obtain a classifier that can analyse wheat spike regions using the standard deep-learning approach, with little knowledge of wheat spike dimensional and spatial characteristics. We fulfilled this requirement by establishing an FCN model to segment spike regions in wheat growth image series acquired in three consecutive years, with varied weather conditions. The spike regions in all images have been annotated at pixel level by specialists using an annotating tool [66]. The model performance was verified on both validation (the 2016 image set) and testing (the 2017 image set) datasets. We have found that FCN was relatively successful at detecting the spike regions in both 2016 (MA: 82.13%) and 2017 (MA: 76.0%). In addition, FCN performed better when trained on larger subimages sizes. We then applied transfer learning to improve the performance of our FCN model by loading parameters learned from ImageNet, and this has led to a positive impact on the segmentation results. The limitations of our research can be summarised by three points: (1) the model had limited success when identifying spike regions in booting and heading; this may be caused by a lack of training data at the two stages; (2) the model encountered some unexpected background objects such as grass, and this has increased false positive rates; again, we believe that more training data or data augmentation could resolve this issue; (3) the model performed relatively poorly on the 2017 set due to challenging lighting and weather conditions. We might be able to overcome some of these image-based limitations by including more historic or artificial images in the training set as well as exploring other deep-learning segmentation architectures such as DeepLap [67] and also some traditional ML segmentation methods. We will also trial other learning tasks in a multitask learning environment to improve the soundness of the solution.

## Figures and Tables

**Table 1:** FCN training hyperparameters.

Stage	Hyperparameter	Value
Initialisation	Weights	(i) He et al. [55] (scratch)
		(ii) ImageNet (transfer)
	Bias	0

Dropout	Rate *p*	0.5

Intermediate Non Linearity Unit	ReLu	

Epochs	125 – 150	

Optimisation (SGD)	Learning Rate	0.001
	Momentum	0.9
	Decay	0.0016
	Mini Batch	20

**Table 2:** Quantitative results of segmentation performance for the 2016 dataset when training FCN from scratch by initialising the weights using He et al.'s method [55] and by loading pretrained ImageNet parameters showing different evaluation metrics.

*Initialisation*	GA	MA	Spike Accuracy	MIoU	Spike IoU
He et al. [55]	92.4	80.14	64.3	70.0	48.02

ImageNet Parameters	*93.54*	*82.13*	*67.55*	*73.0*	*53.0*

**Table 3:** Quantitative results of segmentation performance for the 2017 dataset when training FCN from scratch by initialising the weights using He et al.'s method and by loading pre-trained ImageNet parameters showing different evaluation metrics.

*Initilisation*	GA	MA	Spike Accuracy	MIoU	Spike IoU
He et al. [55]	88.18	70.30	46.61	59.4	31.76

ImageNet Parameters	*90.12*	*76.0*	*57.0*	*64.30*	*40.0*

**Table 4:** Quantitative results of segmentation performance for the 2016 dataset when training FCN with two different subimage size *S* (i.e., 128×128 and 512×512) showing different evaluation scores.

*S*	GA	MA	Spike Accuracy	MIoU	Spike IoU
128×128	93.15	76.1	54.0	70.32	46.1

512 × 512	*93.54*	*82.13*	*67.55*	*73.0*	*53.0*

**Table 5:** Quantitative results of segmentation performance for the 2017 dataset when training FCN with two different subimage size *S* (i.e., 128×128 and 512×512) showing different evaluation metrics scores.

*S*	GA	MA	Spike Accuracy	MIoU	Spike IoU
128×128	90.0	67.02	37.0	60.0	30.1

512 × 512	*90.12*	*76.0*	*57.0*	*64.30*	*40.0*

**Table 6:** Quantitative results of segmentation performance of FCN for the 2016 dataset reported for each growth stage.

Growth Stage	GA	MA	Spike Accuracy	MIoU	Spike IoU
Late booting (GS45-47)	97.41	67.6	37.3	55.0	12.33

Heading (GS51-59)	92.72	77.01	59.2	62.0	31.0

Flowering (GS61-69)	93.30	84.0	70.3	77.0	61.0

Grain filling (GS71-73)	94.0	87.12	77.14	80.14	67.53

Mean	*93.54*	*82.13*	*67.55*	*73.0*	*53.0*

**Table 7:** Quantitative results of segmentation performance of FCN for 2017 dataset reported for each growth stage.

Growth Stage	GA	MA	Spike Accuracy	MIoU	Spike IoU
Middle/late booting (GS43-47)	93.22	60.75	28.0	49.0	3.0

Heading (GS51-59)	91.3	77.7	61.1	64.1	37.4

Flowering (GS61-69)	89.0	80.0	66.0	69.4	51.14

Grain filling (GS71-73)	88.24	80.0	55.03	68.0	50.0

Mean	*90.12*	*76.0*	*57.0*	*64.30*	*40.0*

## Data Availability

The dataset supporting the results is available at https://github.com/tanh86/ws_seg/tree/master/CQ, which includes source code and other supporting data in the GitHub repository.

## Abbreviations

CNNs: Convolutional neural networks
DL: Deep learning
ML: Machine learning
ReLU: Rectified linear units
UK: The United Kingdom.

## Additional Points

*Availability and Requirements. *Operating system(s): Platform independent. Programming language: Python 3. Requirements: TensorFlow, Keras, NumPy, Scikit-image, and OpenCV 3.x.

## Supplementary Materials

Supplementary Materials Supplementary Figure 1: the target traits of the segmentation.
