# Supplementary figures and images for: An Exploration of Deep-Learning Based Phenotypic Analysis to Detect Spike Regions in Field Conditions for UK Bread Wheat
Source: Plant Phenomics. 2019 Jul 31;2019:7368761. doi: 10.34133/2019/7368761 (PMC7706304; doi:10.34133/2019/7368761)

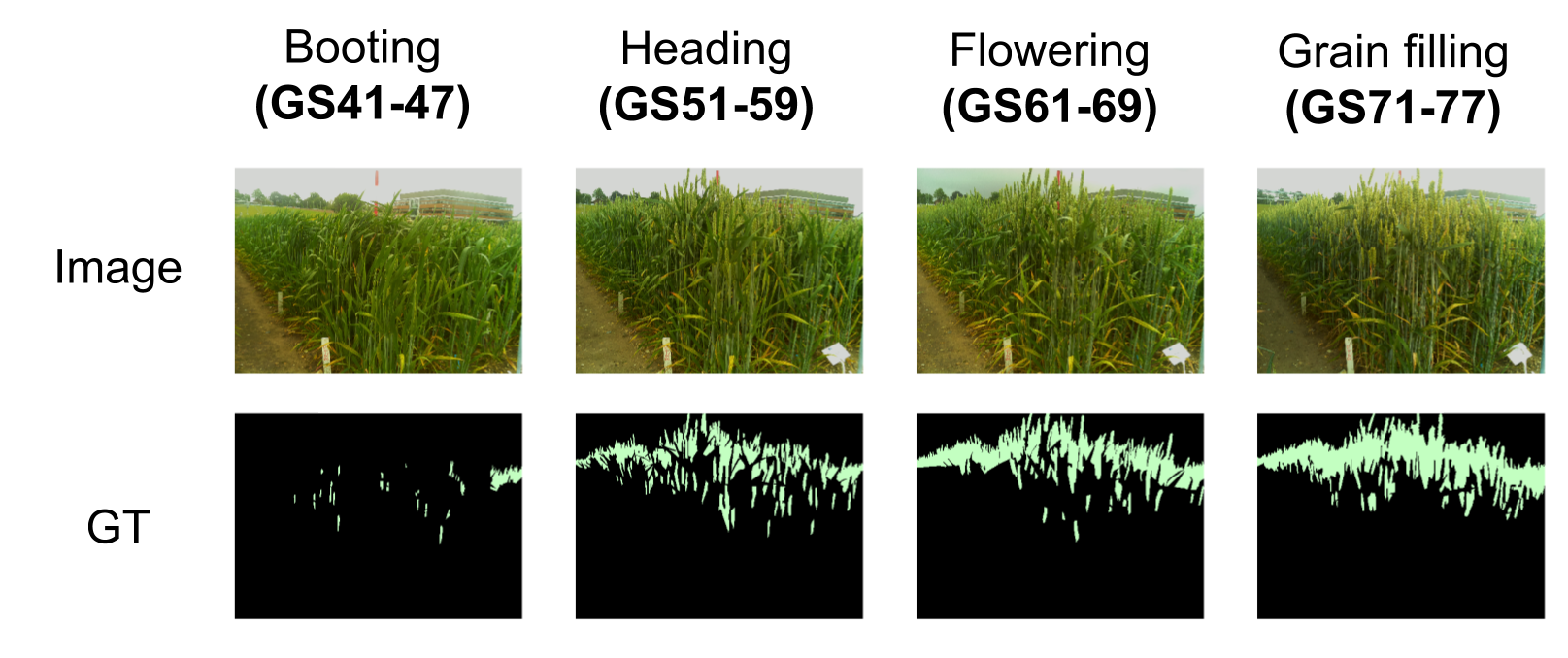

Supplement: Supplementary Materials — Supplementary Figure 1: the target traits of the segmentation. [file 7368761.f1.png]
